# The Effectiveness of Inodilators in Reducing Short Term Mortality among Patient with Severe Cardiogenic Shock: A Propensity-Based Analysis

**DOI:** 10.1371/journal.pone.0071659

**Published:** 2013-08-15

**Authors:** Romain Pirracchio, Jiri Parenica, Matthieu Resche Rigon, Sylvie Chevret, Jindrich Spinar, Jiri Jarkovsky, Faiez Zannad, François Alla, Alexandre Mebazaa, for the GREAT network

**Affiliations:** 1 Department of Anesthesiology and Critical Care Medicine, Hôpital Européen Georges Pompidou, Paris, France; 2 Department of Biostatistics, INSERM UMR-S717, Hôpital Saint Louis, Paris, France; 3 Internal Cardiology Department, Faculty Hospital Brno, ICRC and Medical Faculty of Masaryk University, Brno, Czech Republic; 4 Department of Anesthesiology & Critical Care Medicine, INSERM UMR-S942, Hôpital Lariboisière, Paris, France; 5 Department of Cardiology, University Hospital of Nancy, Nancy, France; 6 Department of Epidemiology, University Hospital of Nancy, Nancy, France; The Ohio State University, United States of America

## Abstract

**Background:**

The best catecholamine regimen for cardiogenic shock has been poorly evaluated. When a vasopressor is required to treat patients with the most severe form of cardiogenic shock, whether inodilators should be added or whether inopressors can be used alone has not been established. The purpose of this study was to compare the impact of these two strategies on short-term mortality in patients with severe cardiogenic shocks.

**Methods and Results:**

Three observational cohorts of patients with decompensated heart failure were pooled to comprise a total of 1,272 patients with cardiogenic shocks. Of these 1,272 patients, 988 were considered to be severe because they required a vasopressor during the first 24 hours. We developed a propensity-score (PS) model to predict the individual probability of receiving one of the two regimens (inopressors alone or a combination) conditionally on baseline-measured covariates. The benefit of the treatment regimen on the mortality rate was estimated by fitting a weighted Cox regression model. A total of 643 patients (65.1%) died within the first 30 days (inopressors alone: 293 (72.0%); inopressors and inodilators: 350 (60.0%)). After PS weighting, we observed that the use of an inopressor plus an inodilator was associated with an improved short-term mortality (HR: 0.66 [0.55–0.80]) compared to inopressors alone.

**Conclusions:**

In the most severe forms of cardiogenic shock where a vasopressor is immediately required, adding an inodilator may improve short-term mortality. This result should be confirmed in a randomized, controlled trial.

## Introduction

Cardiogenic shock, which is characterized by inadequate tissue perfusion in the presence of cardiac failure, is one of the leading causes of hemodynamic instability in critically ill patients and is associated with a high mortality rate [1]. Hemodynamic resuscitation aims at optimizing organ perfusion and avoiding the evolution toward multiple organ failure. Optimized organ perfusion relies on a cautious, limited volume fluid challenge that is usually followed, in the case of persistent instability, by the administration of inotropes [2]. Because blood pressure occasionally fails to increase, vasopressors are often considered as the next-step therapy [2]. However, vasopressors might alter peripheral microcirculation [3] and increase left ventricular afterload [4]. Hence, two theories are encountered: 1) treatment should focus on inotropic support and limitation of left ventricular afterload to avoid any type of vasoconstriction [5]; and 2) similar to sepsis, cardiogenic shock is rapidly associated with a systemic inflammatory state that may require additional vasopressors to restore organ perfusion [6].

The most frequently used vasopressors are norepinephrine, epinephrine and high-dose dopamine [7], [8]. All of these agents are *inopressors* because they interact to different degrees with both the alpha-adrenergic and beta-adrenergic receptors [9]. However, if the gain in terms of inotropic activity is overweighed by the increase in left ventricular afterload, the resultant cardiac output may be decreased and organ perfusion may be further compromised. Levy et al. [10] randomly compared epinephrine to dobutamine plus norepinephrine in a small sample of patients (n = 30) with cardiogenic shock. They showed that the global hemodynamic effects of the two strategies were similar; however, regional perfusion parameters, such as gastric mucosa perfusion, may be improved by combined use of norepinephrine and dobutamine.

The question of the best drug regimen in patients with severe cardiogenic shock must be resolved. It is unclear whether inodilators should be combined with inopressors when the latter are needed or whether inopressors can be used alone. In practice, this question is frequently encountered when a patient fails to improve although dobutamine has been administered. Should we add norepinephrine to dobutamine or switch to epinephrine and discontinue the dobutamine infusion? This question most likely justifies the need for a large multicenter, randomized, controlled trial. However, before designing such a trial, we should explore the data derived from observational studies. Therefore, the goal of this study was to assess, using a propensity-based analysis of observational data, whether the initial regimen of pharmacological hemodynamic therapy could impact short-term mortality in patients with cardiogenic shock who require an inopressor.

## Analysis

### Study Design

The study relies on propensity-score (PS) analyses of three pooled observational datasets. The reporting follows the checklist proposed in the STROBE statement [11].

### Cohorts and Data Sources

The data analyzed in this observational study were derived from three registries of patients with acute heart failure: the ALARM-HF cohort [8], the EFICA cohort [7] and the Czech registry of acute heart failure – AHEAD [12]. Patients with cardiogenic shock after a successfully resuscitated cardiac arrest were not considered.

The *ALARM-HF global survey* (Acute Heart Failure Global Registry of Standard Treatment) collected anonymous data from 4,953 patients originating from nine countries: France, Germany, Italy, Spain, the United Kingdom, Greece, Turkey, Australia, and Mexico [8]. The hospital sample was selected to represent various categories relevant to geographic region, hospital size (number of beds), sector (public vs. private) and type (university vs. nonteaching status). The study was conducted as a retrospective, in-hospital, observational survey via a questionnaire. The paper-based data collection was conducted from October 2006 to March 2007. The same patient was not allowed to be represented more than once. Acute heart failure was the final diagnosis for all of the studied patients based on the 2005 ESC/ESICM guidelines [13]. We collected the type and the severity of cardiac decompensation, and we identified patients who were classified as cardiogenic shock (n = 520).

The *EFICA (Etude Française de l’Insuffisance Cardiaque Aigue)* study is an observational follow-up study of patients with severe acute decompensated heart failure (excluding acute coronary syndromes) who were admitted to a representative sample of 60 French units randomly selected from a national registry of adult intensive care units (ICUs) and coronary care units (approximately 500) in public, semiprivate and private hospitals in France, which commonly admit patients with severe heart failure [7]. The purpose of this study was to investigate the clinical and etiologic features of severe heart failure; to measure the impact of disease in terms of length of hospital stay and of short- and long-term mortality rates; and to evaluate the determinants of short- and long-term survival. From April to October 2001, the 60 participating centers (18 university hospitals, 31 general hospitals and 11 semiprivate and private hospitals) included 599 patients, of whom 152 were considered to be patients with cardiogenic shock.

The *Czech Acute Heart Failure Database (AHEAD) registry* was created to assess the basic characteristics, management and outcome of patients with acute heart failure in the Czech Republic [12]. The anonymous data from 674 patients with consecutive cardiogenic shock were collected from September 2006 to October 2009. The AHEAD registry included consecutive patients from seven centers with a 24-hour catheterization laboratory service and centralized care for patients with acute coronary syndromes (ACS) from a region of approximately three million inhabitants, and from five regional hospitals without a catheterization laboratory service. The participating centers were chosen to represent various categories relevant to geographic region and hospital type (university vs. nonteaching status). Cardiogenic shock was also defined according to the 2005 ESC/ESICM guidelines [13].

### Ethical Issues

The studies were considered to be retrospective patient record studies in which the patients and doctors participating were anonymous prior to processing the data. The studies were conducted according to the principles of the Declaration of Helsinki and approved by national ethics committees (Comité Consultatif sur le Traitement de l'Information en Matière de Recherche, Comité d’Ethique pour la Recherche Biomédicale, Ethics Committee of the Faculty Hospital Brno), which delivered a waiver of informed consent. Additionally, the data treatment was conducted in accordance with the ICC/ESOMAR code of conduct governing market research, specifically the compliance with the “safe harbor” rules and US HIPAA Privacy rule. Concerning the EFICA cohort, a national review board (Comité consultatif sur le traitement de l’information en matière de recherche, French Ministry of Research) approved the study.

### Definition for Cardiogenic Shock

The definition used for cardiogenic shock in the ALARM-HF study and the AHEAD registry was the one proposed in the 2005 ESC/ESICM guidelines [13]: evidence of tissue hypoperfusion induced by heart failure after correction of preload, characterized by a reduced blood pressure (systolic BP<90 mm Hg or a drop of mean arterial pressure >30 mm Hg) and a low urine output (<0.5 ml/kg/h), with a pulse rate >60 b.p.m. with or without evidence of organ congestion. Because of the different time periods, the criteria used for EFICA were slightly different. However, in the EFICA study, all of the patients’ charts were reviewed for the diagnosis of acute decompensated heart failure by a steering committee composed of five senior cardiologists, two senior intensive care specialists and three epidemiologists. Patients who received assist devices and extracorporeal circulation and postcardiac surgical patients were not included in the study.

**Figure 1:**
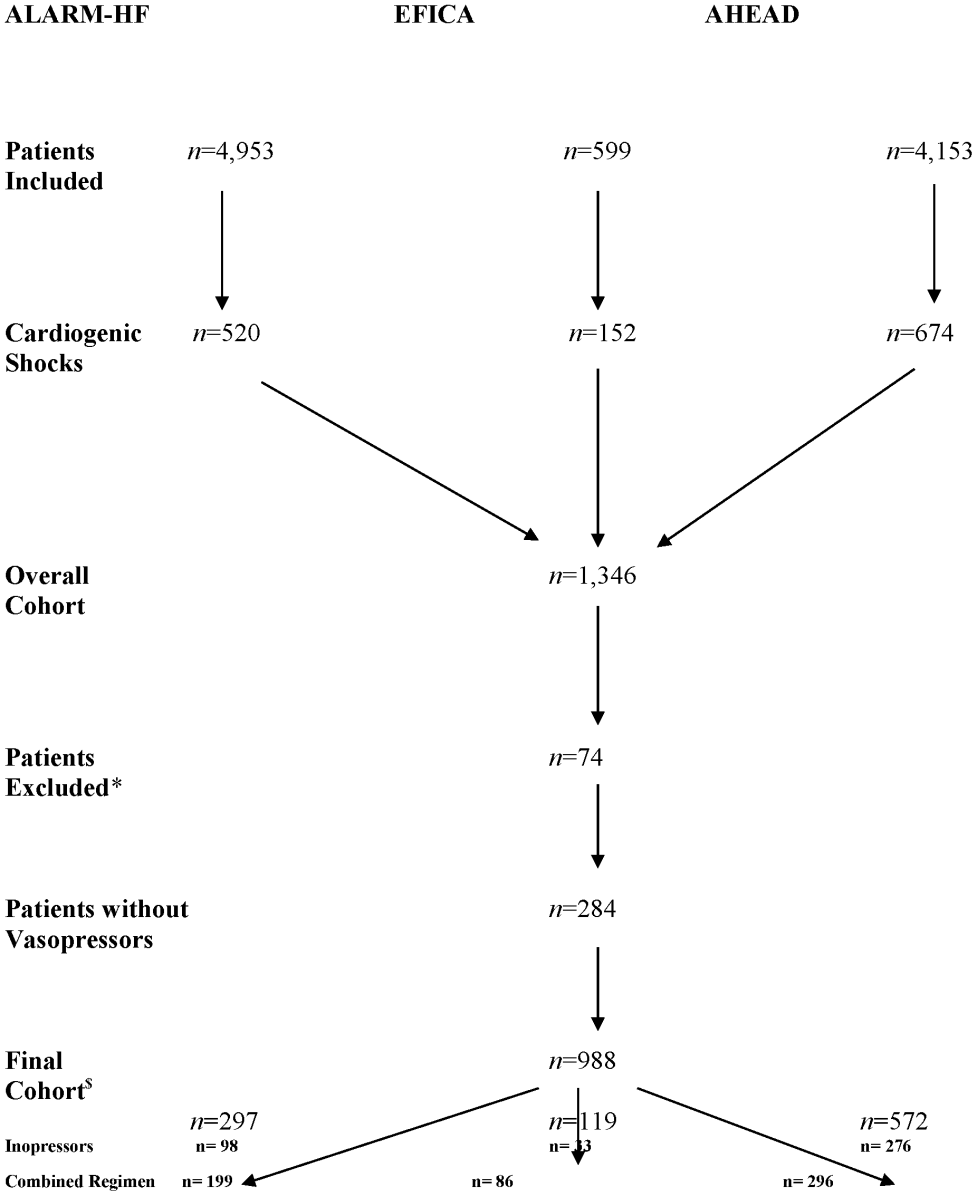
Flowchart. Combined regimen stands for *inopressors and inodilators*; *patients excluded for missing data concerning the treatment regimen, the outcome or the length of hospital stay; ^$^patients included in the final analysis.

Pooling the three studies led to a dataset of 1,346 patients with cardiogenic shock. A total of 74 patients (5.5%) presenting one or more missing data in the treatment regimen or outcome were excluded from the analysis, leading to a total of 1,272 patients. Among those patients, we focused on the 988 patients who required an inopressor during the first 24 hours to achieve the hemodynamic goals (Figure 1).

For each cohort, the number of patients was slightly different from those previously published. Concerning the AHEAD cohort, previous publications involved the main registry (two biggest centers) [12], whereas we used the global registry. Concerning the EFICA and the ALARM-HF cohorts, we excluded several patients because of missing data in the treatment regimen or outcome.

### Patient Evaluation

The following potential confounders or effect modifiers were recorded in the three studies and included our analysis: age; gender; New York Heart Association Classification (NYHA); first systolic blood pressure (SBP); first heart rate (HR); first B-type plasma natriuretic peptide concentration (BNP); first left ventricular ejection fraction (LVEF); first serum creatinine concentration (serum creatinine); acute coronary syndrome (ACS); history of renal disease (renal disease), diabetes mellitus, coronary artery disease (CAD) or chronic heart failure (CHF); teaching hospital; treatment with continuous positive airway pressure (CPAP); and the presence of a prior cardiovascular treatment (beta blockers, renin-angiotensin inhibitors, diuretics, and nitrates). Details of the intravenous drugs administered for AHF, including timing and duration, were also registered. For most variables, the proportion of missing data for other variables was less than 10%. For missing data concerning variables other than the treatment regimen, the length of stay or the outcome, a multiple imputation procedure was applied.

### Drug Regimens

The following drugs were identified in the dataset: epinephrine, norepinephrine, dopamine, dobutamine, levosimendan and phosphodiesterase III inhibitors. We classified these drugs according to their action on the vascular tone. Hence, we differentiated two different types of drugs: the *inodilators*, i.e., drugs associated with inotropic and vasodilator activities (dobutamine, levosimendan and phosphodiesterase III inhibitors), and the *inopressors*, i.e., drugs associated with inotropic and inopressor activities (epinephrine, norepinephrine, and dopamine). The distribution of the initial drug regimen was homogeneous across countries. In all of the studies, the drugs were administered using continuous infusion.

The primary goal of the study was to investigate the effects of treating cardiogenic shock patients with *inopressors alone* or with *inopressors and inodilators* on short-term mortality. We defined and compared the two regimens as follows: the *inopressors alone* and the *inopressors and inodilators* (association of at least one inodilator with an inopressor). Patients who received an inodilator alone were not considered for the present analysis because they were usually less severe than those who required an inopressor. In this study, we focused on the initial drug regimen, corresponding to the drugs received during the first 24 hours after the diagnosis of cardiogenic shock.

### Primary Outcome Measure

The patients were followed from their first day of admission until day 30. The primary outcome measure was mortality up to day 30. None of the 988 patients included in the final analysis presented missing data on the primary outcome measure.

### Statistical Methods

Continuous variables are presented as the mean (standard deviation) or median (interquartile range) as appropriate, whereas the categorical variables are presented as the number (percentage).

Because of the nonrandomized design, the two treatment groups could not be considered to be exchangeable. The distribution of baseline risk factors may have differed between the groups, and several of them may bias the relationship between the treatment and the outcome. To address this problem, we used a propensity score approach [14]. The probability to receive either *inopressors alone* or *inopressors and inodilators* was modeled using a nonparsimonious (including all of the potential confounders/effect modifiers) logistic regression model. The PS model calibration (Hosmer-Lemeshow goodness-of-fit test) and discrimination performance (area under the receiver operating curve) are provided in Appendix S2. The individual propensity score derived from this model was then used in an inverse probability of treatment weighted (IPTW) analysis [15].

Mortality was modeled using Cox proportional hazards model. Administrative censoring was applied on day 30 after admission. Individual propensity scores were used to derive individual weights defined as the inverse of the probability of receiving the treatment actually received and used to weight the Cox regression [15]. The balance in the baseline risk factors was evaluated in the original and the weighted population by computing the standardized differences (SD) [15]. The adequate balance was considered to be achieved for standardized differences below 10% [16].

We included the participants with incomplete data in the analysis using multiple imputations by chained equations with 30 imputations obtained after 10 iterations [17]. For all of the variables except BNP and LVEF, the missing rate was less than 10%. The variables considered in the imputation models were all baseline covariates, death status and Nelson-Aalen estimator of the cumulative hazard at the time of death or censoring [18]. Treatment effect estimates and their standard errors were obtained by pooling the estimates obtained on each imputed dataset according to Rubin’s rules [19].

Several sensitivity analyses were performed. *First*, to evaluate the impact of the missing data and the multiple imputation procedure, we compared the results to those of a complete case analysis. Because these two variables presented a higher rate of missing values, we first excluded the BNP and the LVEF from the propensity score model. We subsequently computed a complete case analysis omitting these two variables. Therefore, all of the patients presenting missing data on any of the variables used in the propensity score model (other than BNP and LVEF) were excluded from the analysis. This exclusion reduced the sample size from 988 to 580. *Second*, we reran the analysis in the subgroups of patients with and without acute coronary syndrome. *Third*, we found 87 patients who received norepinephrine alone, which is not usual in cardiogenic shock. We also reran the analysis after discarding those patients. Finally, we reran the analysis in each cohort separately.

The hazard ratios are expressed together with their 95% confidence intervals. All tests were two-sided at the 0.05 significance level. We performed all of the analyses using R software version 2.13 (http://www.R-project.org) running on a Windows XP platform.

## Results

### Patient’s Characteristics

Pooling the 3 datasets led to an overall population of 1,346 patients with cardiogenic shock. Among these patients, 74 patients were excluded for missing data concerning the treatment regimen, the outcome or the length of hospital stay (Figure 1). Of the remaining 1,272 patients, we focused on the most severe patients, that is, 988 patients who required an inopressor during the first 24 hours. Their characteristics are summarized in Table 1.

**Table 1:** Patients’ characteristics and differences between the two treatment groups.

	Overall	Inopressors Alone	Inopressors and Inodilators	p
	n = 988	n = 407	n = 581	
**Patients’ Characteristics**				
**Age Categories**				0.048
≤45	42	9	33	
46–60	169	70	99	
61–70	252	105	148	
71–80	312	125	187	
>80	212	98	114	
**Gender** (male, %)	632 (64)	238 (58)	394 (68)	0.003
**History**				
**NYHA** (I/II/III/IV)	292/267/225/204	130/120/92/65	167/147/133/139	0.029
**CHF** (%)	306 (31)	101 (25)	205 (35)	<0.001
**CAD** (%)	570 (58)	228 (56)	342 (59)	0.475
**Renal Disease** (%)	259 (26)	78 (19)	181 (31)	<0.001
**Diabetes Mellitus** (%)	396 (40)	150 (37)	246 (42)	0.104
**Prior Treatment** (%)				0.5211 0.443 0.839
Beta Blockers	299 (30)	115 (28)	184 (32)	
ACEI/ARB	404 (41)	166 (41)	238 (41)	
Diuretics	365 (37)	142 (35)	223 (38)	
Nitrates	158 (16)	60 (15)	98 (17)	
**Characteristics at Baseline**				
				
**SBP** (mm Hg)	95 [80–120]	95 [80–120]	95 [80–120]	0.426
**HR** (bpm)	100 [80–120]	93 [75–120]	100 [80–120]	0.111
**BNP** (pg/ml)	1009 [356–3228]	611 [314–2358]	1280 [420–3475]	0.476
**Serum Creatinine** (mg/dl)	1.5 [1.1–2.1]	1.5 [1.1–2.1]	1.5 [1.1–2.1]	0.666
**LVEF** (%)	30 [23–40]	35 [25–45]	30 [20–40]	<0.001
**Cause of Cardiogenic Shock**				
**ACS** (%)	614 (62)	262 (64)	352 (61)	0.254
**Treatment**				
**Inotropes/Vasoactive drugs** (%)				-
Epinephrine	464 (47)	234 (57)	230 (40)	
Norepinephrine	611 (62)	251 (62)	360 (62)	
Dopamine	384 (39)	142 (35)	242 (42)	
Dobutamine	442 (45)	0 (0)	442 (76)	
Levosimendan	96 (10)	0 (0)	96 (16)	
Phosphodiesterase 3 Inhibitor	8 (1)	0 (0)	8 (1)	
**CPAP** (%)	155 (16)	63 (15)	92 (16)	0.950
**Mechanical Ventilation (%)**	567 (57)	234 (57)	333 (57)	0.714
**Primary PCI (%)**	317 (32)	130 (32)	187 (33)	0.957
**Teaching Hospital** (%)	711 (72)	295 (72)	416 (72)	0.817

NYHA, New York Heart Association; ACS, acute coronary syndrome; CAD, coronary artery disease; CHF, chronic heart failure; BNP, B-type natriuretic peptide; renal disease, history of chronic renal failure; CPAP, continuous positive airway pressure; PCI, percutaneous coronary intervention ACEI/ARB, angiotensin-converting-enzyme inhibitors/angiotensin II receptor blockers. The systolic blood pressure (SBP), the heart rate (HR), the BNP, the serum creatinine and the left ventricular ejection fraction (LVEF) are presented as the median [IQR]. For categorical variables, the sum of the different categories might be inferior to the sample size because patients’ characteristics were analyzed from complete cases. The p values refer to the comparison of *inopressors alone* vs. *inopressors and inodilators*.

The patients were essentially male (632 males, 64%) and older than 60 years of age (n = 776, 78%). Before the acute episode, approximately 30% of the patients were classified as NYHA grade 1 (n = 292, 29%), whereas the others were homogeneously distributed over the other 3 grades of the classification. Approximately half of the population (n = 570, 58%) presented an underlying coronary artery disease; the most frequent reason for cardiogenic shock was an acute coronary syndrome (n = 614, 62%). The patients were hypotensive (median systolic blood pressure, 95 mmHg [80–120]) and tachycardic (median heart rate: 100 beats per minute (bpm) [80–120]). In most of the patients, the B-type natriuretic peptide was elevated with a median of 1,009 pg/ml [356–3,228] and the left ventricular ejected fraction was seriously altered (30% [23–40]). Patients’ characteristics according to the cohort of origin are summarized in Appendix S1.

**Figure 2:**
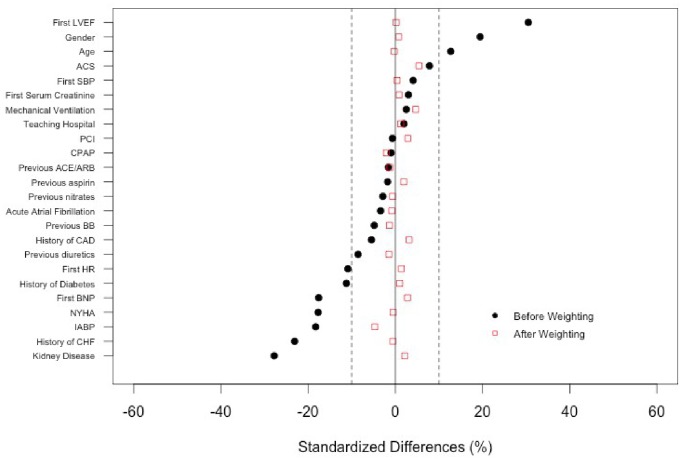
Standard differences in the major baseline covariates between the two treatment regimens. NYHA, New York Heart Association; PCI, percutaneous intervention; IABP, intra-aortic balloon pump; CPAP, continuous positive airway pressure; HR, heart rate; BNP, B-type natriuretic peptide; ACS, acute coronary syndrome; CAD, coronary artery disease; CHF, chronic heart failure; ACEI/ARB, angiotensin-converting-enzyme inhibitors/angiotensin II receptor blockers; BB, beta blockers; SBP, systolic blood pressure; LVEF, left ventricular ejection fraction.

As a result of the nonrandomized design, the patients who received *inopressors alone* were different from those who received *inopressors and inodilators* with respect to baseline characteristics. As illustrated by the standardized differences (Figure 2), those differences were particularly important concerning the history of kidney disease (SD = −27.8%), the history of chronic heart failure (SD = −23.1%), the treatment with IABP (SD = −18.3), gender (SD = 19.4%) or the first LVEF (SD = 30.5%). However, after weighting by the propensity score, all of the standardized differences decreased below the 10% threshold, which suggested that the propensity score weighting adequately handled the initial selection bias (Figure 2 and Table 2).

**Table 2:** Patients’ characteristics and differences between the two treatment groups after PS weighting.

	Inopressors Alone	Inopressors and Inodilators	p
	n = 407	n = 581	
**Patients’ Characteristics**			
**Age Categories (%)** ≤4546–60 61–70 71–80>80	4.917.5 24.1 31.9 21.5	2.8 17.7 27.1 31.9 19.7	0.90
**Gender** (male, %)	36.3	37.2	0.81
**History**			
**NYHA** (I/II/III/IV) (%)	30/27/20/22	29/28/24/18	0.87
**CHF** (%)	31.0	30.7	0.93
**CAD** (%)	57.6	60.1	0.48
**Renal Disease** (%)	26.3	26.9	0.86
**Diabetes Mellitus** (%)	40.3	41.5	0.75
**Prior Treatment** (%) Beta Blockers ACEI/ARB DiureticsNitrates	30.2 40.3 36.3 16.1	30.0 40.4 36.4 16.3	0.94 0.97 0.97 0.95
**Characteristics at Baseline**			
**SBP** (mm Hg)	102 (32)	102 (33)	0.82
**HR** (bpm)	99 (30)	100 (32)	0.64
**BNP** (pg/ml)	2322 (2651)	2475 (2826)	0.50
**Serum Creatinine** (mg/dl)	1.9 (1.7)	1.9 (1.4)	0.88
**LVEF** (%)	33 (15)	33.4 (13.4)	0.97
**Cause of Cardiogenic Shock**			
**ACS** (%)	62.5	65.2	0.44
**Treatment**			
**CPAP** (%)	15.8	14.4	0.56
**Mechanical Ventilation (%)**	57.0	58.9	0.59
**Primary PCI (%)**	31.8	33.6	0.62
**Teaching Hospital** (%)	71.7	72.6	0.77

NYHA, New York Heart Association; ACS, acute coronary syndrome; CAD, coronary artery disease; CHF, chronic heart failure; BNP, B-type natriuretic peptide; renal disease, history of chronic renal failure; CPAP, continuous positive airway pressure; PCI, percutaneous coronary intervention ACEI/ARB, angiotensin-converting-enzyme inhibitors/angiotensin II receptor blockers. The systolic blood pressure (SBP), the heart rate (HR), the BNP, the serum creatinine and the left ventricular ejection fraction (LVEF) are presented as the median [IQR]. For categorical variables, the sum of the different categories might be inferior to the sample size because patients’ characteristics were analyzed from complete cases. The p values refer to the comparison of *inopressors alone* vs. *inopressors and inodilators*.

### Patients’ Outcomes

**Figure 3:**
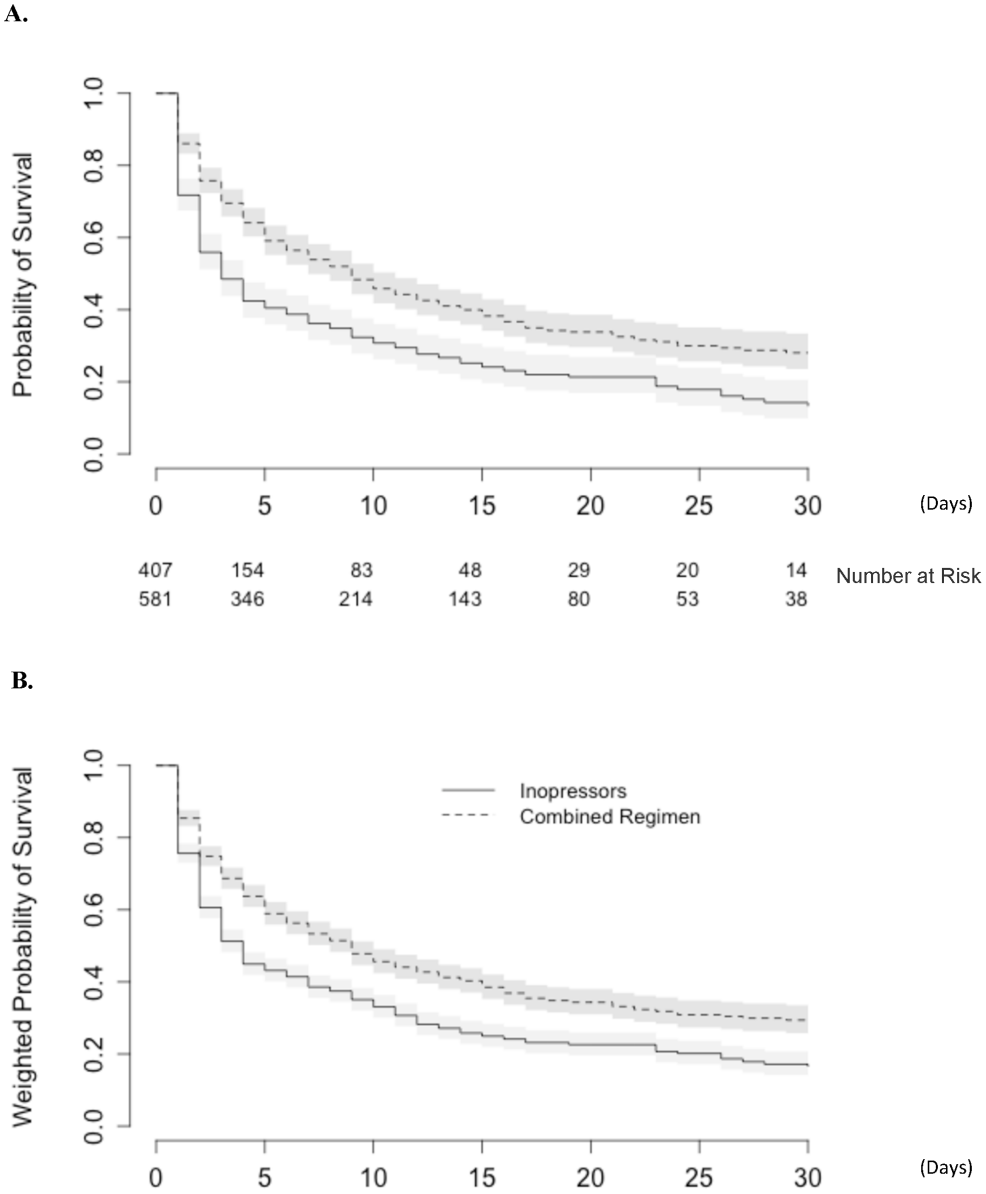
Kaplan-Meier representation of mortality: A. As evaluated in the original pooled datasets; **B.** as evaluated in the pooled datasets after PS weighting. (Combined regimen stands for *inopressors and inodilators*).

All 988 patients were followed up to day 30. A total of 643 patients (65%) died within the first 30 days: 73 patients (61%) in the EFICA cohort, 424 patients (74%) in the AHEAD cohort and 146 patients (49%) in the ALARM-HF cohort. The effect of the initial drug regimen on the original dataset was first studied without any adjustment. The mortality rate highly differed according to the treatment regimens: *inopressors alone*: 293 deaths (72.0%) *vs. inopressors and inodilators*: 350 deaths (60.0%). Consistently, the latter regimen was found to be associated with a decrease in short-term mortality compared to the inopressors alone regimen (HR: 0.61 [0.52–0.71]) (Figure 3, panel A).

The effect of the initial drug regimen on the mortality rate was subsequently assessed among the weighted dataset. The details on the propensity score model are provided in Appendix S2. After weighting by the propensity score, the initial drug regimen was strongly associated with short-term mortality; the regimen of *inopressors and inodilators* performed better than the *inopressors alone* regimen (HR: 0.66 [0.55–0.80]) (Figure 3, panel B).

### Sensitivity Analyses

We reran the analyses after removing the two variables with the higher rate of missing data from the propensity score model, namely, the initial left ventricular ejection fraction and the initial BNP. The results were not substantially modified. The regimen *inopressors and inodilators* was associated with a decrease in short-term mortality compared to the inopressors regimen (HR: 0.70 [0.59–0.83]). In the complete case analysis (n = 580), similar results in terms of mortality were observed when we compared the *inopressors and inodilators* regimen to the *inopressors alone* regimen (HR: 0.69 [0.56–0.86]).

**Figure 4:**
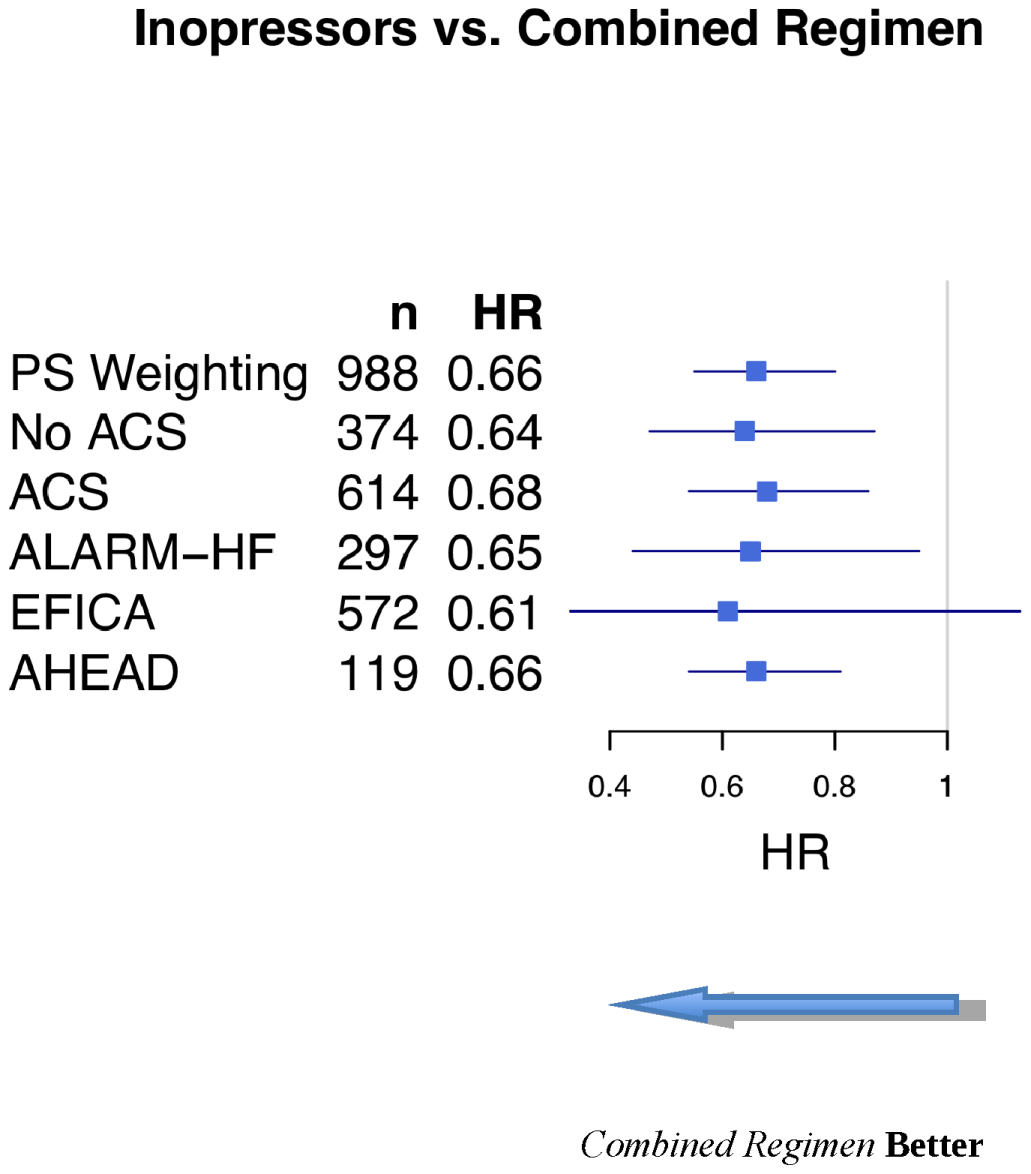
Subgroup PS-weighted analyses of the *inopressors and inodilators* vs. *inopressors alone* on short-term mortality. HR, hazard ratios; PS, propensity score; SBP, systolic blood pressure; ACS, acute coronary syndrome.

As shown in Figure 4, the results remained unaltered when we focused on the subgroups of patients with or without an acute coronary syndrome (Figure 4). Similarly, discarding the 87 patients who received norepinephrine alone did not further modify our results (*inopressors and inodilators vs. inopressors alone*: HR: 0.60 [0.49–0.73]). The results remained unaltered when we removed the patients who received epinephrine alone (HR: 0.70 [0.54–0.92]).

Finally, the results remained unchanged when we analyzed each cohort separately (Figure 4), except when the EFICA cohort was analyzed alone. In the latter case, because of the small sample size, the point estimate was similar but the confidence interval crossed the unit.

## Discussion

The impact of using inopressors in cardiogenic shock is still open to discussion. In the sickest patients, blood pressure may fail to increase after initial resuscitation (e.g., fluid challenge and inotropes), prompting the use of an inopressor, as proposed by the current international guidelines [2]. Inopressors may be justified because prolonged hypotension could precipitate organ hypoperfusion, and especially decreased coronary perfusion pressure, thereby increasing the risk of myocardial ischemia. Moreover, a recent cardiogenic shock paradigm suggested that severe impairments in cardiac function may be associated with systemic inflammation and thus with a certain degree of vasodilatation [6]. Inopressors might, in turn, alter peripheral microcirculation [3], increase left ventricular afterload [4] and thus alter organ perfusion. We used the data from 3 different observational cohorts (ALARM-HF [8], EFICA [7] and AHEAD [12]) and compared inopressors alone to a combination of inopressors plus inodilators. We found that using inopressors alone could be associated with a greater short-term mortality compared to using a combined regimen. This finding would suggest either that excessive vasoconstriction is detrimental in patients with severe cardiogenic shock or that vasodilation is beneficial. Because of the nonrandomized design, we cannot conclude that the patients who received inopressors alone were sicker than those who received *inopressors and inodilators*. However, the patients’ characteristics were well balanced after propensity score weighting, and IPTW analysis led to similar results.

Vasoconstriction *per se* could be considered to be potentially harmful in this context. Excessive vasoconstriction may, in turn, impair macro-hemodynamics by increasing left ventricular afterload [4], [20] and promoting myocardial oxygen delivery/oxygen consumption imbalance [21]. Catecholamines have been advocated to affect the restoration of cardiac function and increase short- and long-term mortalities [22]. Excessive vasoconstriction might also alter microcirculation [3]. Such a hypothesis has been well documented in the context of septic shock [23], [24]. In cardiogenic shock, there are few data [25]; however, microvascular blood flow alterations are reportedly frequent and more severe in patients who are not likely to survive [26]. However, the detrimental effect of vasoconstriction might not be the only explanation to support our results. The trial by De Backer et al. [27] showed that a drug with a strong inotropic effect (dopamine) could be deleterious compared to a potent vasopressor with a weak inotropic activity (norepinephrine).

Vasodilation *per se* could be considered to be beneficial in this context. Vasodilatation might preserve the microcirculation, organ perfusion and likely the patient’s outcome. Such a hypothesis is supported by the fact that applying low-dose nitroglycerin, in addition to standard care for cardiogenic shock, has been shown to result in an increase in sublingual perfused capillary density [28]. Our results are also consistent with the findings recently reported by Levy et al. [10], which suggest that a combination of norepinephrine-dobutamine might be more reliable and safer than epinephrine in patients with cardiogenic shock. In their entire cohort, Spinar et al. [12] reported an association between norepinephrine infusion and in-hospital mortality. However, inopressors are recommended and usually used because the hemodynamic goals fail to be reached with the use of inotropes alone. Combining our results with those of De Backer et al. [27] would suggest that inopressors, such as norepinephrine, might be necessary in severe patients, but the addition of a vasodilator might be beneficial. These results might be considered to be conflicting compared to several previous studies [29]. These conflicting results could be explained by the differences in the study populations. For instance, in OPTIME-CHF [30], the patients in shock were excluded, whereas in the ADHERE registry [31], less than 3% of the patients had low systolic blood pressure. Moreover, the question we addressed was different. We focused on estimating the benefit of adding a drug with vasodilator-related properties to inopressors. Therefore, all of the patients in our cohort received inopressors, which makes this population more severe. This point is illustrated by the higher mortality rate in our cohort: On day 30, the mortality rate was 65% in our cohort, whereas it was 10.3% in the milrinone group and 8.9% in the control group in OPTIME-CHF, and 11% in the ICU/CCU group in the ADHERE registry.

Our study suffers several limitations. *First*, the lack of randomization exposed to selection bias is a limitation. To handle such a risk, we used a propensity score weighting approach (IPTW) [15]. *Second*, several of the measured variables had a high rate of missing data. We applied a multiple imputation algorithm and controlled the results by performing a complete case analysis. *Third*, we pooled various drugs to define the different treatment regimens. However, two drugs defined as inopressors can have a very different pharmacodynamic profile. Our goal was to assess the impact of vasoconstriction on the prognosis; and this goal could only be achieved by comparing the drugs with and without inopressor activity. We were also concerned about the fact that norepinephrine and epinephrine might have different effects on patients with cardiogenic shock because epinephrine is a more potent inotrope than norepinephrine. However, removing from the analysis the patients who received norepinephrine alone did not alter our results. *Fourth*, the enrollment period of the three cohorts was long. However, studies on shock, either observational or interventional, are difficult to perform and usually require long inclusion periods. For instance, De Backer et al. included 1,679 shock patients in a 4-year period to complete their study [27]. Moreover, the guidelines for the management of cardiogenic shock were not substantially modified during the last decade, and our results were similar when we analyzed separately the older and the more recent cohorts. *Fifth*, the definition of cardiogenic shock was slightly different between EFICA and the other two registries. However, in the EFICA study, all of the patients’ charts were reviewed for the diagnosis of acute decompensated heart failure by a steering committee; the diagnostic criteria that were applied did not substantially change over the last decade. Moreover, the results were consistent when analyzing the three cohorts separately. *Sixth*, the systolic blood pressure could be considered to be surprisingly high. However, tissue hypoperfusion, rather than systolic blood pressure, is the cornerstone of the diagnosis of shock [2]. Hence, the mean blood pressure levels reported by De Backer et al. [27] or Levy et al. [10] were 58 mm Hg and 55 mm Hg, respectively, which is consistent with a systolic blood pressure of approximately 90 mm Hg. *Seventh*, we did not record the drug dose and were therefore unable to assess the presence of dose and response relationships, which is essentially a problem for dopamine, whose effects on vascular tones are dosage-dependent. To further explore this potential source of bias, we performed a complementary sensitivity analysis, which showed similar results after excluding those patients who received dopamine (HR: 0.63 [0.50–0.80]). *Finally*, we only focused on initial treatment and clinical parameters. We were unable to analyze the whole treatment history or the evolution of hemodynamic profile under treatment. Additional studies that consider the entire ICU stay will therefore be warranted.

Our results may have important clinical implications. Our findings support the concept that cardiogenic shock requires a certain degree of vasodilation, although the macro-hemodynamics prompt the use of vasopressors. Hence, in such a situation, we should most likely prefer adding inopressors to inodilators and monitor the results in terms of cardiac output and venous oxygen saturation for a better optimization.

### Conclusion

The initial use of *inopressors alone* appears to be associated with a poorer prognosis compared to a regimen of *inopressors and inodilators* in patients who are admitted for cardiogenic shock and who require an inopressor during the first 24 hours. When an inopressor is needed to achieve hemodynamic goals in patients with cardiogenic shock, combining a drug with a vasodilating activity might be useful. This result, based on observational data, emphasizes the urgent need for a large multicenter, randomized, controlled trial to compare those two regimens.

## Supporting Information

Appendix S1 **Patients’ characteristics according to the cohort.**
(DOCX)

Appendix S2 **Propensity Score Model.** Calibration as evaluated by the Hosmer-Lemeshow statistic: X-squared = 8.91; p = 0.444; discrimination as evaluated by the area under the receiver operating curve: AUC-ROC = 0.700.
(DOC)

## References

[1] Krenn L, Delle Karth G (2011) Essential lessons in cardiogenic shock: epinephrine versus norepinephrine/dobutamine. Crit Care Med 39: 583–584 doi:10.1097/CCM.0b013e318208e381 21330855 10.1097/CCM.0b013e318208e381

[2] McMurray JJV, Adamopoulos S, Anker SD, Auricchio A, Böhm M, et al (2012) ESC Guidelines for the diagnosis and treatment of acute and chronic heart failure 2012: The Task Force for the Diagnosis and Treatment of Acute and Chronic Heart Failure 2012 of the European Society of Cardiology. Developed in collaboration with the Heart Failure Association (HFA) of the ESC. Eur Heart J 33: 1787–1847 doi:10.1093/eurheartj/ehs104 22611136 10.1093/eurheartj/ehs104

[3] Krejci V, Hiltebrand LB, Sigurdsson GH (2006) Effects of epinephrine, norepinephrine, and phenylephrine on microcirculatory blood flow in the gastrointestinal tract in sepsis. Crit Care Med 34: 1456–1463 doi:10.1097/01.CCM.0000215834.48023.57 16557162 10.1097/01.CCM.0000215834.48023.57

[4] Thompson RB, van den Bos EJ, Esposito DJ, Owen CH, Glower DD (2003) The effects of acute afterload change on systolic ventricular function in conscious dogs with normal vs. failing hearts. Eur J Heart Fail 5: 741–749. 14675852 10.1016/s1388-9842(03)00152-1

[5] Capomolla S, Pozzoli M, Opasich C, Febo O, Riccardi G, et al (1997) Dobutamine and nitroprusside infusion in patients with severe congestive heart failure: hemodynamic improvement by discordant effects on mitral regurgitation, left atrial function, and ventricular function. Am Heart J 134: 1089–1098. 9424070 10.1016/s0002-8703(97)70030-9

[6] Hochman JS (2003) Cardiogenic shock complicating acute myocardial infarction: expanding the paradigm. Circulation 107: 2998–3002 doi:10.1161/01.CIR.0000075927.67673.F2 12821585 10.1161/01.CIR.0000075927.67673.F2

[7] Zannad F, Mebazaa A, Juillière Y, Cohen-Solal A, Guize L, et al (2006) Clinical profile, contemporary management and one-year mortality in patients with severe acute heart failure syndromes: The EFICA study. Eur J Heart Fail 8: 697–705 doi:10.1016/j.ejheart.2006.01.001 16516552 10.1016/j.ejheart.2006.01.001

[8] Mebazaa A, Parissis J, Porcher R, Gayat E, Nikolaou M, et al (2011) Short-term survival by treatment among patients hospitalized with acute heart failure: the global ALARM-HF registry using propensity scoring methods. Intensive Care Med 37: 290–301 doi:10.1007/s00134-010-2073-4 21086112 10.1007/s00134-010-2073-4

[9] How O-J, Røsner A, Kildal AB, Stenberg TA, Gjessing PF, et al (2010) Dobutamine-norepinephrine, but not vasopressin, restores the ventriculoarterial matching in experimental cardiogenic shock. Transl Res J Lab Clin Med 156: 273–281 doi:10.1016/j.trsl.2010.07.011 10.1016/j.trsl.2010.07.011 20970750

[10] Levy B, Perez P, Perny J, Thivilier C, Gerard A (2011) Comparison of norepinephrine-dobutamine to epinephrine for hemodynamics, lactate metabolism, and organ function variables in cardiogenic shock. A prospective, randomized pilot study. Crit Care Med 39: 450–455 doi:10.1097/CCM.0b013e3181ffe0eb 21037469 10.1097/CCM.0b013e3181ffe0eb

[11] Von Elm E, Altman DG, Egger M, Pocock SJ, Gøtzsche PC, et al (2007) The Strengthening the Reporting of Observational Studies in Epidemiology (STROBE) statement: guidelines for reporting observational studies. Plos Med 4: e296 doi:10.1371/journal.pmed.0040296 17941714 10.1371/journal.pmed.0040296 PMC2020495

[12] Spinar J, Parenica J, Vitovec J, Widimsky P, Linhart A, et al (2011) Baseline characteristics and hospital mortality in the Acute Heart Failure Database (AHEAD) Main registry. Crit Care Lond Engl 15: R291 doi:10.1186/cc10584 10.1186/cc10584 PMC3388663 22152228

[13] Nieminen MS, Böhm M, Cowie MR, Drexler H, Filippatos GS, et al (2005) Executive summary of the guidelines on the diagnosis and treatment of acute heart failure: the Task Force on Acute Heart Failure of the European Society of Cardiology. Eur Heart J 26: 384–416 doi:10.1093/eurheartj/ehi044 15681577 10.1093/eurheartj/ehi044

[14] Paul R Rosenbaum, Rubin DB (1983) The central role of the propensity score in observational studies for causal effects. Biometrika 70: 41–55.

[15] Hernán MA, Brumback B, Robins JM (2000) Marginal structural models to estimate the causal effect of zidovudine on the survival of HIV-positive men. Epidemiol Camb Mass 11: 561–570. 10.1097/00001648-200009000-00012 10955409

[16] Austin PC (2009) Balance diagnostics for comparing the distribution of baseline covariates between treatment groups in propensity-score matched samples. Stat Med 28: 3083–3107 doi:10.1002/sim.3697 19757444 10.1002/sim.3697 PMC3472075

[17] White IR, Kalaitzaki E, Thompson SG (2011) Allowing for missing outcome data and incomplete uptake of randomised interventions, with application to an Internet-based alcohol trial. Stat Med 30: 3192–3207 doi:10.1002/sim.4360 21948462 10.1002/sim.4360 PMC3279649

[18] White IR, Royston P (2009) Imputing missing covariate values for the Cox model. Stat Med 28: 1982–1998 doi:10.1002/sim.3618 19452569 10.1002/sim.3618 PMC2998703

[19] Rubin DB (1996) Multiple Imputation After 18+ Years. J Am Stat Assoc 91: 473–489.

[20] Heusch G (1990) Alpha-adrenergic mechanisms in myocardial ischemia. Circulation 81: 1–13. 1967557 10.1161/01.cir.81.1.1

[21] Nikolaidis LA, Hentosz T, Doverspike A, Huerbin R, Stolarski C, et al (2002) Catecholamine stimulation is associated with impaired myocardial O(2) utilization in heart failure. Cardiovasc Res 53: 392–404. 11827690 10.1016/s0008-6363(01)00490-4

[22] Reynolds HR, Hochman JS (2008) Cardiogenic shock: current concepts and improving outcomes. Circulation 117: 686–697 doi:10.1161/CIRCULATIONAHA.106.613596 18250279 10.1161/CIRCULATIONAHA.106.613596

[23] Palizas F, Dubin A, Regueira T, Bruhn A, Knobel E, et al (2009) Gastric tonometry versus cardiac index as resuscitation goals in septic shock: a multicenter, randomized, controlled trial. Crit Care Lond Engl 13: R44 doi:10.1186/cc7767 10.1186/cc7767 PMC2689488 19335912

[24] Boerma EC, Koopmans M, Konijn A, Kaiferova K, Bakker AJ, et al (2010) Effects of nitroglycerin on sublingual microcirculatory blood flow in patients with severe sepsis/septic shock after a strict resuscitation protocol: a double-blind randomized placebo controlled trial. Crit Care Med 38: 93–100 doi:10.1097/CCM.0b013e3181b02fc1 19730258 10.1097/CCM.0b013e3181b02fc1

[25] Jung C, Rödiger C, Fritzenwanger M, Schumm J, Lauten A, et al (2009) Acute microflow changes after stop and restart of intra-aortic balloon pump in cardiogenic shock. Clin Res Cardiol Off J Ger Card Soc 98: 469–475 doi:10.1007/s00392-009-0018-0 10.1007/s00392-009-0018-0 19367424

[26] De Backer D, Creteur J, Dubois MJ, Sakr Y, Vincent JL (2004) Microvascular alterations in patients with acute severe heart failure and cardiogenic shock. Am Heart J 147: 91–99. 14691425 10.1016/j.ahj.2003.07.006

[27] De Backer D, Biston P, Devriendt J, Madl C, Chochrad D, et al (2010) Comparison of dopamine and norepinephrine in the treatment of shock. N Engl J Med 362: 779–789 doi:10.1056/NEJMoa0907118 20200382 10.1056/NEJMoa0907118

[28] Den Uil CA, Lagrand WK, Spronk PE, van der Ent M, Jewbali LSD, et al (2009) Low-dose nitroglycerin improves microcirculation in hospitalized patients with acute heart failure. Eur J Heart Fail 11: 386–390 doi:10.1093/eurjhf/hfp021 19211570 10.1093/eurjhf/hfp021

[29] Bayram M, De Luca L, Massie MB, Gheorghiade M (2005) Reassessment of dobutamine, dopamine, and milrinone in the management of acute heart failure syndromes. Am J Cardiol 96: 47G–58G doi:10.1016/j.amjcard.2005.07.021 10.1016/j.amjcard.2005.07.021 16181823

[30] Cuffe MS, Califf RM, Adams KF Jr, Benza R, Bourge R, et al (2002) Short-term intravenous milrinone for acute exacerbation of chronic heart failure: a randomized controlled trial. Jama J Am Med Assoc 287: 1541–1547. 10.1001/jama.287.12.1541 11911756

[31] Adams KF Jr, Fonarow GC, Emerman CL, LeJemtel TH, Costanzo MR, et al (2005) Characteristics and outcomes of patients hospitalized for heart failure in the United States: rationale, design, and preliminary observations from the first 100,000 cases in the Acute Decompensated Heart Failure National Registry (ADHERE). Am Heart J 149: 209–216 doi:10.1016/j.ahj.2004.08.005 15846257 10.1016/j.ahj.2004.08.005

