# Correction: The Effectiveness of Inodilators in Reducing Short Term Mortality among Patient with Severe Cardiogenic Shock: A Propensity-Based Analysis

**DOI:** 10.1371/annotation/add6bc4f-dec0-4c95-a609-67563469b831

**Published:** 2013-12-10

**Authors:** Romain Pirracchio, Jiri Parenica, Matthieu Resche Rigon, Sylvie Chevret, Jindrich Spinar, Jiri Jarkovsky, Faiez Zannad, François Alla, Alexandre Mebazaa, for the GREAT network

The "Combined Regimen" values in Figure 1 were erroneously altered. Please see the corrected Figure 1 here: http://plosone.org/corrections/pone.0071659.g001.cn.tif

The P-Values in Table 1 were erroneously altered. Please see the corrected Table 1 here: http://plosone.org/corrections/pone.0071659.t001.cn.tif

