# Supplementary material for: The Effectiveness of Inodilators in Reducing Short Term Mortality among Patient with Severe Cardiogenic Shock: A Propensity-Based Analysis
Source: PLoS One. 2013 Aug 15;8(8):e71659. doi: 10.1371/journal.pone.0071659 (PMC3744474; doi:10.1371/journal.pone.0071659)
Supplement: Appendix S1 — Patients’ characteristics according to the cohort. (DOCX) [file pone.0071659.s001.docx]

|  | **AHEAD** | **EFICA** | **ALARM-HF** |
| --- | --- | --- | --- |
|  | n=572 | n=119 | n=297 |
| **Patients’ Characteristics** |  |  |  |
| **Age Categories**  ≤ 45  46-60  61-70  71-80  >80 | 13  90  134  180  155 | 8  17  28  43  23 | 21  60  91  89  34 |
| **Gender** (male, %) | 346 (60) | 77 (65) | 209 (70) |
| **History** |  |  |  |
| **NYHA** (I/II/III/IV) | 174/181/125/30 | 44/32/38/5 | 38/28/46/146 |
| **CHF** (%) | 173 (30) | 75 (63) | 58 (19) |
| **CAD** (%) | 385 (67) | 52 (44) | 132 (44) |
| **Renal Disease** (%) | 93 (16) | 49 (41) | 116 (39) |
| **Diabetes Mellitus** (%) | 225 (39) | 33 (28) | 138 (46) |
| **Prior Treatment** (%)  Beta Blockers  ACEI/ARB  Diuretics  Nitrates | 200 (35)  71 (24)  42 (14)  98 (17) | 26 (22)  55 (46)  68 (57)  27 (23) | 35 (12)  238 (41)  223 (38)  17 (6) |
| **Characteristics at Baseline** |  |  |  |
| **SBP** (mm Hg) | 105 [85-130] | 90 [73-106] | 85 [75-10] |
| **HR** (bpm) | 90 [76-110] | 104 [86-120] | 110 [90-128] |
| **BNP** (pg/ml) | 1192 [591-2165] | NA | 2100 [1138-3160] |
| **Serum Creatinine** (mg/dl) | 1.5 [1.1-2.0] | 1.9 [1.5-2.6] | 1.3 [1.0-2.0] |
| **LVEF** (%) | 30 [23-40] | 30 [20-40] | 30 [20-40] |
| **Cause of Cardiogenic Shock** |  |  |  |
| **ACS** (%) | 374 (65) | 55 (46) | 185 (62) |
| **Treatment** |  |  |  |
| **Inotropes/Vasoactive Drugs** (%)  Epinephrine  Norepinephrine  Dopamine  Dobutamine  Levosimendan  Phosphodiesterase 3 Inhibitor | 303 (53)  494 (86)  155 (27)  218(38)  66 (11)  0 (0) | 75 (63)  33 (28)  53 (44)  84 (71)  0 (0)  2 (2) | 86 (29)  84 (28)  176 (59)  140 (47)  30 (10)  6 (2) |
| **CPAP** (%) | 102 (18) | 12 (10) | 41 (14) |
| **Mechanical Ventilation (%)** | 334 (58) | 91 (76) | 133 (45) |
| **Primary PCI (%)** | 229 (40) | 4 (3) | 82 (28) |
| **Teaching Hospital** (%) | 511 (89) | 39 (33) | 161 (54) |

*Appendix S1. Patients’ characteristics according to the cohort.*
