# Supplementary material for: The Effectiveness of Inodilators in Reducing Short Term Mortality among Patient with Severe Cardiogenic Shock: A Propensity-Based Analysis
Source: PLoS One. 2013 Aug 15;8(8):e71659. doi: 10.1371/journal.pone.0071659 (PMC3744474; doi:10.1371/journal.pone.0071659)
Supplement: Appendix S2 — Propensity Score Model. Calibration as evaluated by the Hosmer-Lemeshow statistic: X-squared = 8.91; p = 0.444; discrimination as evaluated by the area under the receiver operating curve: AUC-ROC = 0.700. (DOC) [file pone.0071659.s002.doc]

|  | Coefficients | Standard Error |
| --- | --- | --- |
| *Intercept* | 2.160 | 0.805 |
| *Country* | -0.040 | 0.052 |
| *Cohort*  *AHEAD*  *EFICA* | -0.487  0.397 | 0.320  0.334 |
| *Age* | -0.029 | 0.068 |
| *Gender* | -0.349 | 0.150 |
| *NYHA* | -0.091 | 0.087 |
| *History of CHF* | 0.364 | 0.211 |
| *History of CAD* | -0.0004 | 0.190 |
| *History of Kidney Disease* | 0.554 | 0.177 |
| *History of Diabetes* | 0.244 | 0.155 |
| *First HR* | 0.004 | 0.002 |
| *First SBP* | 0.003 | 0.002 |
| *First BNP* | 0.0001 | 0.00003 |
| *First LVEF* | -0.026 | 0.005 |
| *First Serum Creatinine* | -0.073 | 0.048 |
| *Atrial Fibrillation* | -0.246 | 0.130 |
| *ACS* | -0.312 | 0.170 |
| *AHF as Primary Cause for Hospital Admission* | -0.348 | 0.256 |
| *De Novo/Worsening Symptoms* | 0.272 | 0.215 |
| *PCI* | 0.563 | 0.194 |
| *CPAP Use* | -0.017 | 0.194 |
| *Teaching Hospital* | 0.461 | 0.249 |
| *Hospital Size* | -0.275 | 0.129 |
| *Hospital Type* | -0.198 | 0.143 |

*Appendix S2. Propensity Score Model.* Calibration as evaluated by the Hosmer-Lemeshow statistic: X-squared=8.91; p- =0.444; discrimination as evaluated by the area under the receiver operating curve: AUC-ROC=0.700; p =1.
